# Multimodal Approach of Optical Coherence Tomography and Raman Spectroscopy Can Improve Differentiating Benign and Malignant Skin Tumors in Animal Patients

**DOI:** 10.3390/cancers14122820

**Published:** 2022-06-07

**Authors:** Mindaugas Tamošiūnas, Oskars Čiževskis, Daira Viškere, Mikus Melderis, Uldis Rubins, Blaž Cugmas

**Affiliations:** 1 Biophotonics Laboratory, Institute of Atomic Physics and Spectroscopy, University of Latvia, 19 Raina Blvd., LV-1586 Riga, Latvia; oskars.cizevskis@lu.lv (O.Č.); daira.viskere@llu.lv (D.V.); mikus.melderis@lu.lv (M.M.); uldis.rubins@lu.lv (U.R.); 2 Faculty of Veterinary Medicine, Latvia University of Life Sciences and Technologies, 8 K. Helmana Str., LV-3004 Jelgava, Latvia

**Keywords:** linear discriminant analysis, lipoma, mast cell tumor, multidimensional data, optical coherence tomography, Raman spectroscopy, soft tissue sarcoma, veterinary oncology

## Abstract

**Simple Summary:**

Skin and subcutaneous tumors are among the most frequent neoplasms in dogs and cats. We studied 51 samples of canine and feline skin, lipomas, soft tissue sarcomas, and mast cell tumors using a multimodal approach based on optical coherence tomography and Raman spectroscopy. A supervised machine learning algorithm detected malignant tumors with the sensitivity and specificity of 94% and 98%, respectively. The proposed multimodal algorithm is a novel approach in veterinary oncology that can outperform the existing clinical methods such as the fine-needle aspiration method.

**Abstract:**

As in humans, cancer is one of the leading causes of companion animal mortality. Up to 30% of all canine and feline neoplasms appear on the skin or directly under it. There are only a few available studies that have investigated pet tumors by biophotonics techniques. In this study, we acquired 1115 optical coherence tomography (OCT) images of canine and feline skin, lipomas, soft tissue sarcomas, and mast cell tumors ex vivo, which were subsequently used for automated machine vision analysis. The OCT images were analyzed using a scanning window with a size of 53 × 53 μm. The distributions of the standard deviation, mean, range, and coefficient of variation values were acquired for each image. These distributions were characterized by their mean, standard deviation, and median values, resulting in 12 parameters in total. Additionally, 1002 Raman spectral measurements were made on the same samples, and features were generated by integrating the intensity of the most prominent peaks. Linear discriminant analysis (LDA) was used for sample classification, and sensitivities/specificities were acquired by leave-one-out cross-validation. Three datasets were analyzed—OCT, Raman, and combined. The combined OCT and Raman data enabled the best sample differentiation with the sensitivities of 0.968, 1, and 0.939 and specificities of 0.956, 1, and 0.977 for skin, lipomas, and malignant tumors, respectively. Based on these results, we concluded that the proposed multimodal approach, combining Raman and OCT data, can accurately distinguish between malignant and benign tissues.

## 1. Introduction

Skin and subcutaneous tumors are among the most frequent neoplasms found in companion animals [1,2,3]. In dogs, the most common skin tumors are malignant mast cell tumors (MCT, a relative frequency of ~16.0%), soft tissue sarcomas (STS, 10.9–25.6%), and benign lipomas (8.5–12.5%) [2,3,4]. MCT are cancerous round cell tumors, which arise from the specific white blood cells called mast cells. They are typically skin neoplasms, but occasionally MCT appear on the lymph nodes, spleen, and liver. Feline mast cell tumors also occur, although less often (1.7–6.8%) [4,5]. In cats, both mesenchymal (connective tissue) tumors are more common with the relative frequencies of 17.9–38.7% (STS) and 5.7% (lipomas) [5,6]. Importantly, lipomas’ prevalence is probably significantly higher than reported since these benign tumors are excised and sent for validation only if they grow enough to disturb normal function [4]. On the other hand, feline injection-site sarcomas are likely to contribute to high STS prevalence since they develop on the vaccination site in 1–10 of every 10,000 vaccinated cats [7].

**Figure 1:**
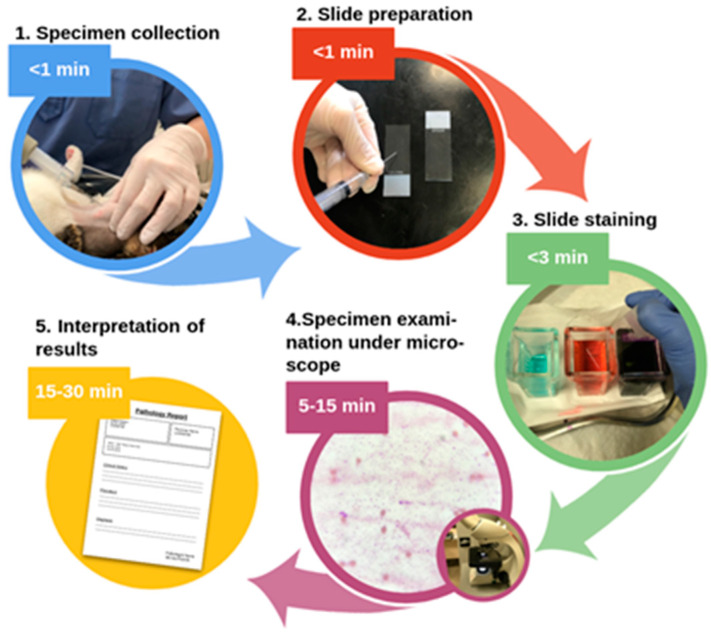
Fine-needle cytology method. (**1**) Specimen collection: cells are collected from the neoplasia; (**2**) slide preparation: aspirated material expelled on the slide, smeared using the second slide, and dried; (**3**) slide staining: slide stained using the Diff-Quik staining method [12]; (**4**) specimen examination under a microscope: veterinary pathologist conducts a visual examination of the stained material; (**5**) interpretation of results: inflammatory process from neoplasia differentiated (when possible), neoplasia classified into 1 of 3 categories (epithelial, mesenchymal, or round cell neoplasia) and the prognostic answer is given (benign tissue or malignant tumor).

Since 20–40% of skin tumors in dogs and 70–80% in cats are malignant [2], clinical veterinarians aim to identify potential cancer promptly. For the initial evaluation, fine-needle cytology (FNC) is generally performed. A thin needle (i.e., typically 22–25 gauge) is inserted into the tumor to harvest cells (Figure 1). The collected cells are placed on a slide, stained, and examined under a microscope. The studies revealed that FNC reached the sensitivity and specificity of between 68.6 and 89.3% and 77.2 and 100%, respectively, against histopathology [4,8,9]. The examiner’s experience, tumor type, and aspiration utilization can also weaken the performance of FNC. An inflammation component can be dominant in cancers such as mammary tumors [10], which makes finding tumorous cells challenging. In addition to lymphoma, these epithelial tumors can even resemble inflammation or benign tumors, leading to a low FNC accuracy of <50% [4,11].

Undoubtedly, early detection of malignant lesions could help facilitate the therapeutic approach and thus the prognosis of the veterinary patient. The physicians need reliable and straightforward diagnostic techniques for canine skin and subcutaneous tumors. Several biophotonic techniques have been proposed to diagnose or characterize canine and feline skin and subcutaneous tumors, providing complementary information to FNC or tissue histological examination. The malignant tumors were detected based on different absorption (i.e., water and hemoglobin concentration) and scattering properties (i.e., reduced scattering coefficient) with an accuracy of between 54.6 and 90.0% [13]. Raman spectroscopy was applied to characterize malignant tissue in canine mammary glands and microcalcifications [14,15]. A direct comparison of Raman spectral data to the histopathology findings helped to identify Raman peaks at 1332 cm^−1^ (CH_2_/CH_3_ vibrations in collagen), 1450 cm^−1^ (CH_2_ bending mode in albumin), and 1453 cm^−1^ (structural modes of proteins) as Raman biomarkers of tissue malignancy [14]. Raman peaks at 960 cm^−1^ and 1070 cm^−1^ have been observed in benign and malignant canine tumors, respectively, due to the accumulation of hydroxyapatite deposits [14]. The Raman spectral band intensity at 1486 cm^−1^ has been reported to be higher in STS and MCT tumors in cats and dogs [16] arising from adenine and guanine nucleobase vibrations. For clinical veterinary applications, the scalpel blade with a gold SERS (surface-enhanced Raman scattering) accessory was tested by Munteanu et al. [17]. Raman spectrum fingerprints of carotenoids and interfacial water discovered in canine solid canine carcinoma and lobular hyperplasia samples indicated promising applications of SERS for tumor margin detection [17].

More research are being conducted for the OCT-based identification of canine and feline cancer. Selmic et al. [18,19,20,21,22,23] showed that optical coherence tomography (OCT) could be a promising tool for guiding small-animal surgeons and pathologists to areas of interest, improving the diagnostic accuracy (67.9–93.3%), and assessing surgical margins. OCT was shown to be appropriate to distinguish between the tissues with different densities (adipocytes, collagen fibers, fibrotic versus oedematous stroma) or cellularity (i.e., multifocal degranulation in MCT or necrotic spots in STS).

The integration of multimodal data in preclinical oncology research is mainly restricted to animal models of cancer [24]. Preclinical research in dogs and cats with naturally existing tumors is in demand as it is more complementary to human cancer since the tumor cells interact with the functioning immune system [25]. The existing veterinary studies were limited to applying biophotonic techniques (Raman or OCT) separately to diagnose the structure or biochemical composition of neoplasms. The present study applied Raman spectroscopy on the samples pre-characterized by OCT, with the aim to improve the accuracy of tumor tissue diagnosis. Multidimensional data were obtained ex vivo from the most common canine and feline skin and subcutaneous tumors: mast cell tumors (MCT), soft tissue sarcomas (STS), and benign lipomas. The diagnostic features were extracted from both OCT images and Raman spectra. Linear discriminant analysis (LDA) was used as a machine learning model for distinguishing between malignant and benign tumors. Our study is an important step towards the optical characterization of small-animal skin and subcutaneous tumors, which could represent an alternative to the current invasive diagnostic methods such as FNC.

## 2. Materials and Methods

The study was conducted under permission No. DZLAEP-2021/3 of the Ethical Council of Animal Welfare and Protection (Latvia University of Life Sciences and Technologies, Jelgava, Latvia). We collected: 13 lipomas, which belonged to 11 dogs and 2 cats; 23 soft tissue sarcomas in 14 dogs and 9 cats; 15 mast cell tumors in 12 dogs and 3 cats. The mean age of animals was 8.5 years (the full range was 2–13 years). More details on sample collection are presented in Table S1, including the definition of species, breed, age, tissue types examined, grade of tumor malignancy, and tumor localization.

After the tumor excision, the samples were put in 10% formalin and sent for histopathology to Academic histology laboratory Ltd. (Riga, Latvia). After tissue dissection to select the appropriate examination areas, the primary treatment of surgical material, embedding in paraffin blocks, slide preparation, and staining with H&E was performed. The slides were digitized with Pannoramic 250 FLASH III DX brightfield slide scanner (3DHistech Kft., Budapest, Hungary) using 20× or 40× magnification. The pathologist assessed the images of each specimen to characterize the sample structure.

For the remaining part of the sample, the pathologist marked the precise tumor location with small pins, put the samples back to the 10% formalin solution, and sent them to our laboratory for OCT and Raman analysis. The skin tissue bordering the tumor was examined as the reference.

Spectral-domain OCT (sd-OCT) images were taken using a commercial Telesto II (Thorlabs, Newton, NJ, USA) device equipped with 1325 nm SLD excitation light source with ~100 nm spectral bandwidth, which enabled to achieve 9 μm axial resolution. The lateral resolution, 7 μm, depended on the Thorlabs telecentric scan-lens used (OCT-LK2, EFL = 18 mm). To guarantee an optical image quality, we acquired images at a 28 kHZ A-scan rate (a dynamic range of 95 dB), kept the beam focus point approximately 1 mm below the tissue surface, and slightly tilted the samples. We generated final *.tiff images by averaging 20 A and 20 B scans. Up to 14 cross-sectional images on each tumor and native skin (as a reference) were taken with 6 mm (lateral) and 3.5 mm (depth) dimensions. In total, 1115 OCT images were acquired ex vivo.

Raman spectra were recorded in the range of 920–2070 cm^−1^ by using iHR320 imaging spectrometer equipped with 1200 g/mm grating and thermoelectrically cooled Syncerity 1024×256-OE CCD camera (Horiba, Kyoto, Japan). A 785 nm diode laser excitation (Cobolt 08-NLD, Hübner Photonics, Kassel, Germany) was focused to 0.14 cm^2^ sample area delivering 40 mW incident power. A fiber bundle probe (comprising of 6 fibers, *ϕ*400 μm, NA = 0.22, Light Guide Optics International, Līvāni, Latvia) was used for Raman signal collection, together with an ultra-steep dichroic mirror (#69–905, Edmund Optics, Barrington, NJ, USA) serving to reflect excitation light and to transmit the Raman Stokes signal. The frontal end of the fiber bundle was placed at a 30 mm distance perpendicular to the examined tissue surface and the distal end with an SMA connector was mounted on 220 F fiber-adapter (Horiba) containing 60 mm and 40 mm focal length lenses with incorporated 785 nm long-pass edge filter (BLP01-785R-25, Semrock, Rochester, NY, USA). The detailed schematic diagram of our Raman spectroscopy setup is presented in [26]. Eight to twelve spectra were collected from each sample, using a 90 s scan time, 50 μm slit width, and full vertical binning, reaching a spectral resolution of 0.17 nm. A Raman spectra library was constructed from 1002 spectra measured from the skin, MCT, STS, and lipomas ex vivo.

**Figure 2:**
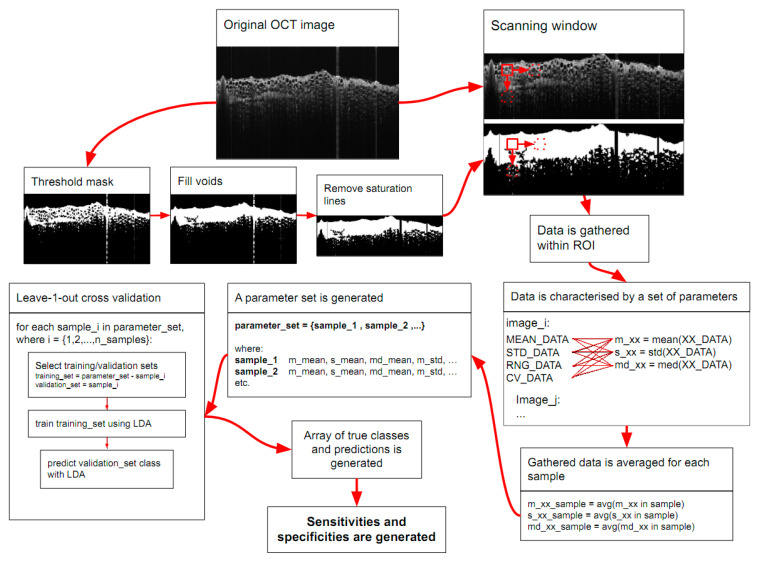
Representative image processing flowchart. An example of a difficult to mask image—lipoma (original OCT image scan region 6 × 3.5 mm). The tissue has many voids in it, and there are strong back reflection lines visible. A naive threshold mask shows why this is an issue. Saturation lines can be mistaken for tissue, and the voids in the tissue structure could be ignored. Hence, the voids were detected and filled, and reflection lines removed. The scanning window, schematically represented by the red square, is moved around within the masked region. The stride is set to 1 in the movement directions (which are indicated by the red arrows). The window looks at each pixel and its surrounding pixels; for a 15 × 15 pixel window, the window contains 225 values. These are then used to calculate μ, σ, intensity range, and CV parameters, and these are then added to an array for later processing. In total, 12 parameters from scanning window data were calculated for each image, then averaged for each sample. The resulting parameter set is used for leave-one-out cross-validation, which generates a table of true and predicted classes. From this, sensitivities and specificities are calculated for each class.

A Python software script (version 3.9.6, Python Software Foundation, Wilmington, DE, US) was written to process the images and analyze the data. The algorithm used the OpenCV library (version 4.5.3, https://opencv.org/ (accessed on 11 May 2022) Open Source Vision Foundation, Palo Alto, CA, USA) for tissue segmentation. The algorithm steps are presented in Figure 2. First, the thresholding served to create the binary mask image (Figure 2, threshold mask). Then, the flood fill method was used to fill the background, resulting in an image where all but the voids are white. This image was then inverted, and pixel values were summed for voids-only image and the original-threshold image, so that the voids had been treated as part of the tissue structure (Figure 2, fill voids).

The saturation line locations were determined by sliding pixels through the x-axis at the very top of the threshold image, which should contain only the background. In practice, it was found that saturation lines were not perfectly straight in the mask image and, due to back reflection effects, might only show up as periodic patches in the y-axis direction. To ensure that all saturation lines were removed, the first 15 rows were taken (denoted as matrix **B**_(15, n)_) and summed according to: **S**_(1,n)_ = **J**_(1,__15__)_ · **B**_(15,n)_, where n is the number of columns and **J**_(1,15)_ is a matrix of ones. If a saturation line is present, the value in the column will be larger than 0. All values in the same column in the full mask image were set to 0 if this was the case, thus excluding the saturation lines from the scanning window analysis. The mask images were rendered and manually checked to ensure all the lines were removed.

After mask generation, the OCT images were analyzed with a 15 × 15 pixel (eq. to ~53 × 53 μm^2^) scanning window with a stride of 1, only collecting data within the masked region (Figure 2, scanning window). For each iteration, we calculated four parameters: μ, σ, intensity range, and coefficient of variation (CV = σ/μ), where σ and μ stand for standard deviation and intensity mean, respectively (in Figure 2 “mean_data; std_data; rng_data; cv_data”). This resulted in 4 distributions per image, one for each parameter. To reduce the number of dimensions for classification, we took mean, standard deviation, and median for each distribution to characterize them in a few parameters (in Figure 2 “m_xx; s_xx; md_xx”, respectively). This resulted in 12 new parameters in total, 3 for each of the four distributions. As up to 14 separate images were acquired per sample, the results were averaged for each sample (in Figure 2 “m_xx_sample; s_xx_sample; md_xx_sample”). The resulting parameters were then used as features for classification.

During Raman spectra preprocessing, the autofluorescence background was eliminated using Labspec 6 software (Horiba, Japan) by fitting 7nd to 9th order polynomial to each spectrum. Spectra were normalized by dividing all Raman intensities with the maximum peak intensity at 1437 cm^−1^ (skin, lipoma) or at 1446 cm^−1^ (MCT, STS). Within the spectral range 920 to 2070 cm^−1^, intensity changes of the Raman spectra between different types of tissues were estimated at: 925 to 970 cm^−1^, 1060 to 1160 cm^−1^, 1180 to 1280 cm^−1^, 1310 to 1380 cm^−1^, 1451 to 1509 cm^−1^, 1845 to 1880 cm^−1^, and 2050 to 2080 cm^−1^ wavenumber intervals. Each new parameter was created based on averaging the normalized Raman spectral intensity values within the wavenumber ranges indicated. This resulted in 7 new parameters extracted from Raman spectral data (p1 to p7). Additionally, the average absolute spectral intensity was used as the 8th parameter (p8).

In total, 3 datasets were created—OCT, Raman, and combined, the latter of which merged both OCT and Raman features into a single data set for each sample. Linear discriminant analysis (LDA, “linearDiscriminantAnalysis” function, in “scikit.learn” library for Python) was used to reduce dimensionality and to classify data in each dataset. The resultant dimension of data was equal to the number of classes minus 1. Sensitivity and specificity values were estimated by using leave-one-out cross-validation, as the number of samples was limited to divide the database into separate training and validation datasets. The way to implement the leave-one-out method was as follows: a single instance (sample_i) was removed from the dataset; the LDA classifier was applied to the remaining data points; the class label of the instance—skin, lipoma, or malignant (STS and MCT) was then compared to the training classification prediction of said instance (Figure 2), resulting in either a match or mismatch. Sensitivity and specificity were calculated according to the leave-one-out cross-validation approach.

Statistical analysis included the unpaired nonparametric Mann–Whitney test to compare the distributions of μ, σ, intensity range, and CV between the tissue classes. This test was also used to determine the significance between the averaged Raman intensity values. Results were considered statistically significant if the p-value was *** *p* < 0.001, ** *p* < 0.01 and * *p* < 0.05; NS—no significant differences.

## 3. Results and Discussion

### 3.1. OCT

**Figure 3:**
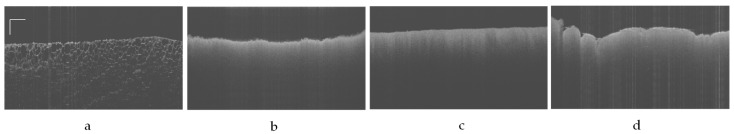
Representative sd-OCT B-scan images of (**a**) lipoma, (**b**) mast cell tumor, (**c**) soft tissue sarcoma, and (**d**) skin. Scale bar: 500 μm.

When studying native OCT images (Figure 3), lipomas exhibited a low reflectance and distinctive honeycomb structure, typical for human adipose tissues [27]. Conversely, OCT images of MCT and STS were signal-dense and highly scattering, thus being more challenging to differentiate. Skin OCT images contained data on different skin layers. The outermost layer (*Stratum corneum*) resembled a highly reflective line, matched with the presence of keratinized cells [28]. Together with the dermal-epidermal junction, the epidermis and dermis exhibited a lower reflectance.

**Figure 4:**
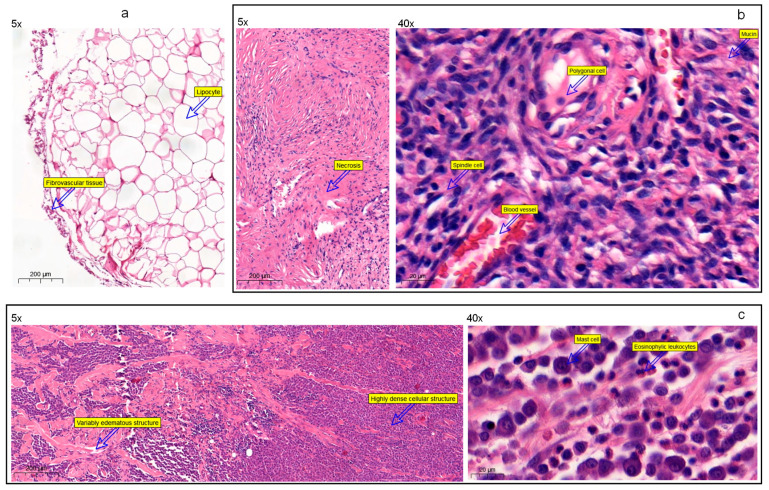
Histological characteristics of lipomas, sarcomas, and mast cell tumors; (**a**) lipoma in a dog, located in subcutis (sample# 20B0847, Table S1). It is a partly delineated, multilobular tumor with a dense cellular structure. The sample includes well-differentiated lipocytes of different sizes—with big fat cell vacuoles and flattened nuclei located in the periphery. The minimal amount of fibrovascular tissues supports tumor cells. (**b**) Soft tissue sarcoma in a dog, found in subcutis (sample# 20B0614, Table S1). It is a well-delineated, unencapsulated, multilobular spindle cell tumor; the cellularity varies from densely to scantly cellular. There are multifocal areas of edema, necrosis, and hemorrhages. Tumor cells are supported by a moderate amount of hyalinized collagen and occasional mucin deposits. Tumor cell morphology is highly variable, ranging from spindle cells—with a small amount of cytoplasm and elongated nucleus with small nucleoli, to polygonal cells—with a moderate amount of cytoplasm and a large, oval nucleus containing 2–4 moderately- to large-sized nucleoli. A prominent proliferation of small blood vessels is in the surrounding tissue. (**c**) Mast cell tumor in a dog, infiltrating dermis and subcutis (sample# 20B0446, Table S1). Wide, nonconfined tumor with highly dense cellular structure surrounded by a moderate amount of variably edematous fibrous stroma. Tumorous tissues are mixed with a moderate to the high amount of eosinophilic leukocytes, making nidus of degranulation in a few places.

The histopathological analysis described lipomas composed of mature adipocytes without atypia (Figure 4a). In one sample, adipocytes lay between striated muscle fibers. We also found atypical adipocytes with multiple mitotic figures, nuclear polymorphism, and infiltrative growth patterns. Sarcomas were characterized as spindle cell tumor fragments with high cellular density, visible mitosis, and atypia (Figure 4b). Tumor cells infiltrated subcutaneous fat tissue, striated muscles, and dermis with significant hemorrhages, edema, tissue necrosis, and stromal hyalinosis. The average sarcoma spindle size was 40 µm, except when giant tumor cells were present in two samples. We also detected the formation of the tumor capsule. Finally, mast cell tumors contained a uniform population of mast cells. In most cases, cellular density was high, and tumor cells infiltrated the dermis or subcutis. Minimal edema and few mitotic figures were found. According to histology findings, mast cells were 16–20 microns in size; however, they appeared in larger clusters and sheets due to the tumor metastasis (Figure 4c).

**Figure 5:**
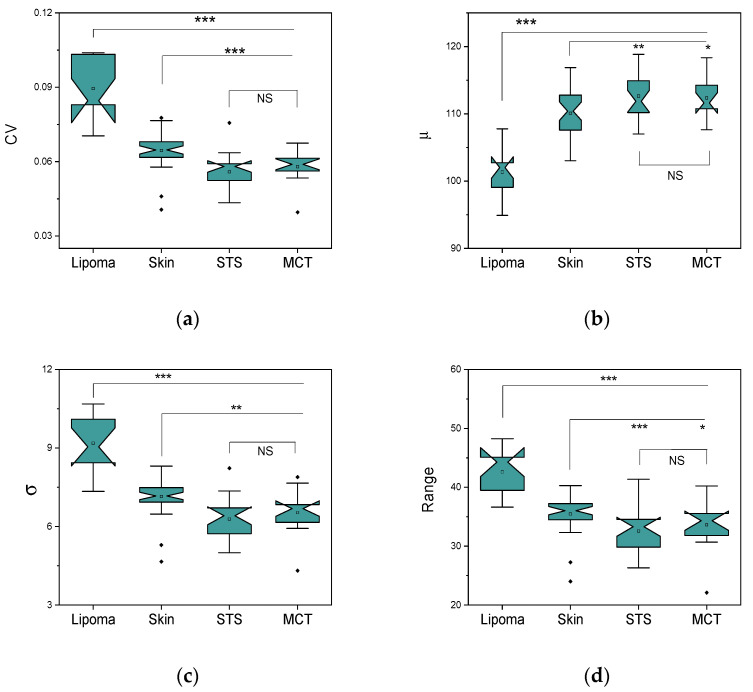
Boxplots of tissue OCT features; (**a**) coefficient of variation (CV), (**b**) intensity mean (μ), (**c**) standard deviation (σ), and (**d**) intensity range. The central mark represents the median; the bottom and top box edges indicate the 25th and 75th percentiles, respectively. Whiskers extend to the most extreme data point. The outliers are marked as dots. Respectively, *p*-values less than 0.05, 0.01, 0.001 are shown by *, **, *** (Mann–Whitney).

Due to their distinct structures in OCT images, lipomas exhibited significantly higher σ, intensity range, and CV parameters than malignant tumors or skin (Figure 5). Expectedly, lipomas had a statistically significant reduction in intensity mean, μ, due to low signal reflectance. The skin samples also displayed some feature variability with a few outliers in the data. The skin tissue was also determined to be statistically different from MCT and STS tumors when the Mann–Whitney test was applied (Figure 5a–d). In contrast, MCT and STS exhibited similar diagnostic parameter values (CV, σ, μ, range) without statistical difference. We also observed that healthy skin showed greater variability and more outliers than malignant tumors (Figure 5a). Smaller CV values tend to appear due to the higher intensity mean. Additionally, a more uniform or homogeneous texture of STS and MCT could result in a minor standard deviation, lowering CV. On the other hand, honeycomb-like adipose tissue and heterogeneous skin exhibited a higher standard deviation, increasing CV. The next measure of variability in the data, range, represents the difference between the extreme pixel intensity values (Figure 5d). This parameter defines the interval of pixel intensity values compared to the full dynamic range. Therefore, standard deviation and range parameters appear correlated. The decision on OCT parameters’ selection was guided by a previous study [18] demonstrating the possibility of applying scanning window analysis to extract parameters from OCT images to classify the tissues. The distributions of σ, intensity range, and CV characterized the variation in the microstructures of the sarcoma, lipoma, and muscle [18].

### 3.2. Raman Spectroscopy

**Figure 6:**
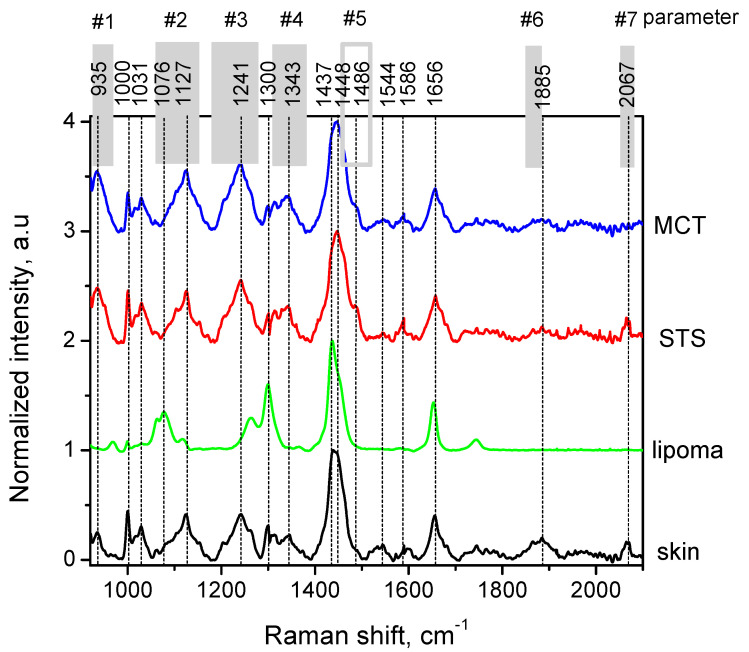
Mean normalized Raman spectra of skin, lipomas, soft tissue sarcomas (STS), and mast cell tumors (MCT). Native skin was present in 31 samples (adjacent to: lipoma—5; MCT—9; STS—17 samples). Gray areas mark the ranges of Raman spectra parameters #1 to #7 (abbreviated as p1—p7). For clarity, an offset of +1 in the y-axis was introduced for each tumor spectrum.

Figure 6 shows the average normalized Raman spectra for all sample classes examined and their major peak assignments in the fingerprint region. Measured Raman spectra consisted of several overlapping and various intensity bands located at 935, 1000, 1031, 1076, 1127, 1241, 1300, 1343, 1437, 1448, 1544, 1586, 1656, 1885, and 2067 cm^−1^. The Raman spectral peaks of skin reported in the literature correspond to dermal protein vibration modes at 935 cm^−1^ (C-C stretching of proline and protein backbone), 1000 cm^−1^ (phenylalanine), 1031 cm^−1^ (carbohydrate residues of collagen), 1127 cm^−1^ (C-N stretching), 1343 cm^−1^ (CH2/CH3 of collagen), 1448 cm^−1^ (CH2/CH3 deformation of collagen), 1544 cm^−1^ (amide II vibration modes), 1586 cm^−1^ (hydroxyproline of collagen), and 1658 cm^−1^ (amide I vibration modes) [29]. The peaks at 1076, 1302, 1437, and 1660 cm^−1^ originate from lipids (mostly triolein) [30] and significantly contribute to the measured spectral bands of lipomas (Figure 6). As mentioned in the literature, the amounts of triolein (estimated as dry weight of total lipids) exceed 99.7% in human lipomas [31], thus validating how lipoma specimens from cats and dogs are being pooled together for automated analysis. Mesa et al. [18] also suggested jointly analyzing the feline and canine tissues (i.e., canine and feline sarcomas were pooled together) since no microstructural variations between species were identified histologically.

Malignant STS and MCT possessed common Raman peaks compared to healthy skin. For example, the Raman peak at 1241 cm^−1^ originates from phosphodiester groups of nucleic acids arising from asymmetric PO^2-^ stretching modes [32]. It highlights the increase in nucleic acids in malignant lesions; however, it overlaps with tertiary amide bands from healthy tissues within the 1220–1250 cm^−1^ spectral region [29]. By further analyzing this region, the Amide III band at 1232 cm^−1^ can also characterize the malignant tissues [33]. The elevated peak shoulder at 1437 cm^−1^ (Figure 6) corresponds to CH2 deformation modes of lipids, indicating either the presence of a benign lesion or normal tissue [34].

Table 1 summarizes the statistical significance between the sample classes for the averaged spectral intervals, indicated as p1–p7 in Figure 6. The construction of the parameters was based on both the literature findings and on actual spectral signatures measured. Other animal studies named the spectral bands peaking at 960 cm^−1^ (included in p1), 1070/1155 cm^−1^ (p2), 1332 cm^−1^ (p4), and 1453/1486 cm^−1^ (p5), thus being informative for cancer type discrimination [14,16,17]. Parameter p1 includes information on microcalcifications occurring in animal tumors. Although microcalcifications have not been observed in MCT and STS tumors histopathologically or in Raman spectra (absence of peaks at 960, 1070, and 1673 cm^−1^), the short wavenumber range holds the potential to deliver more diagnostic value (i.e., for distinguishing MCT vs. skin and STS vs. skin, Table 1). Parameters p2, p3, and p4 have similar diagnostic performance. Despite the good diagnostic performance of p6 and p7, their vibrational frequencies lay in the cell-silent region and are currently undefined.

Calculated average intensity values of the unnormalized Raman spectra have been used as an additional parameter (p8). A *p*-value less than 0.001 was obtained for discrimination between the sample classes (Table 1); however, it should be noted that formalin fixation can produce a decrease in overall Raman spectral intensity due to infiltration into the fixed tissue [35].

### 3.3. Classification

The LDA method was further applied as a supervised learning technique to distinguish between the tissue classes. In total, four classes were used for LDA: skin, lipoma, MCT, and STS. However, since both STS and MCT are malignant tissues, and since STS and MCT features showed little to no statistically significant differences in OCT and Raman, the sensitivity and specificity for a combined MCT + STS malignant tissue class were additionally calculated by cross-referencing sample labels and classifier predictions (Table 2).

**Figure 7:**
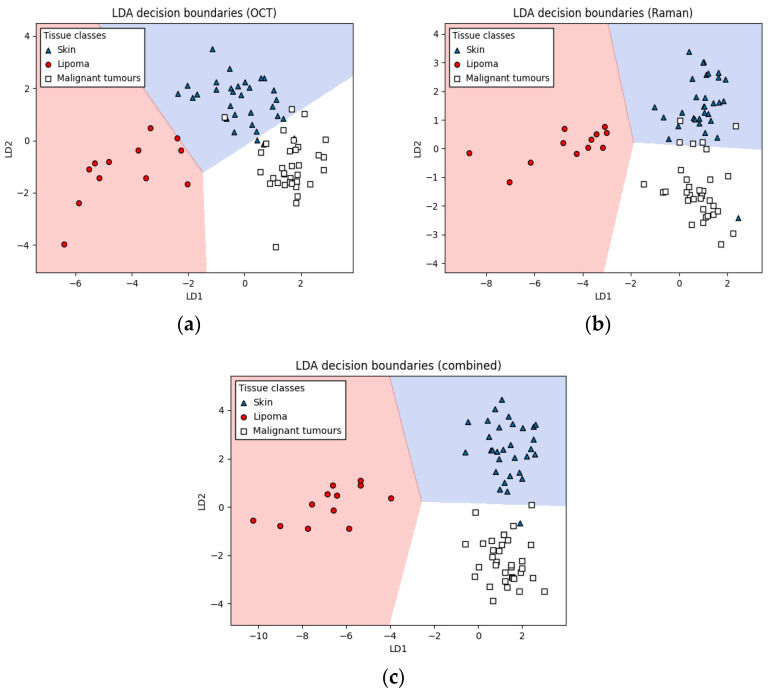
LDA decision boundaries for skin, lipoma, and malignant tumors. Three datasets are shown—OCT (**a**), Raman (**b**), and combined OCT + Raman (**c**). Data are plotted on the two most significant linear discriminants, LD1 and LD2.

The combined OCT–Raman approach achieved malignant tumor detection accuracy between 93.9 and 97.7% (Table 2, Figure 7). Classification based on a single database (OCT or Raman) performed worse, with sensitivities and specificities between 81.8% and 95.3%, respectively. The classification differences can be seen in more considerable distances between the categories of the combined approach (Figure 7c) than in applying OCT and Raman data alone (Figure 7a,b, respectively). In all cases, the lipoma class cluster showed the largest linear separation from other classes, resulting in high classification sensitivities and specificities (i.e., mostly 100%). Similarly, a classifier worked well for skin with the sensitivity and specificity of 0.968 and 0.956, respectively. MCT and STS could not be distinguished very well (sensitivities of 0.786 and 0.737 and specificities of 0.935 and 0.947 for MCT and STS, respectively). However, the combined malignant tumors could be detected very well with a sensitivity of 0.939 and specificity of 0.977, indicating that misclassification occurred mainly between both malignant tissue classes and that the malignant tissue class shows good separation from lipomas and skin.

Our study reached comparable classification accuracies to those in humans, where bladder samples were characterized ex vivo and separately by OCT and Raman spectroscopy [36,37]. Supervised k-nearest neighbor (kNN) and partial least squares linear discriminant analysis (PLS-LDA) classifiers achieved sensitivities and specificities between 60–80% and 68–99% for OCT and Raman datasets, respectively. Both studies find Raman spectroscopy as the preferred method for providing final diagnostic information and OCT as a supporting tool to inspect the microstructure of lesions. Conversely, we did not find OCT data inferior since both datasets produced a similar diagnostic accuracy of around 89%.

Some other human studies [38,39,40,41] demonstrated the advantages of combining the OCT (as a guiding tool) with Raman spectroscopy to acquire tissue structural and biochemical information. However, they mainly focused on being able to image a specific area of the tissue with co-localized Raman and OCT measurements. In contrast, we did not attempt to acquire both OCT and Raman images by co-aligning the diagnostic probes. Sequential OCT and Raman measurements were performed following the pathologist’s decision on tissue morphology in situ and by transferring the sample between the devices operated separately. Our data show that high diagnostic accuracy could be achieved even without the co-localized OCT–Raman measurements.

As in our study, the combined OCT–Raman approach also achieved better tumor classifications in human colon tissues [42]. Separate datasets enabled sensitivities and specificities between 74–89%, but the combined classifier’s accuracy increased to 94%, close to our mean diagnostic accuracy of 95.8% (Table 2). The mentioned study also used a supervised classifier based on the linear support vector machine (SVM) and leave-one-out cross-validation [42]. However, we found that simply using LDA as a classifier resulted in the same classification accuracy [43], so the SVM classifier was removed from our further data analysis algorithm.

We needed up to 25 min to acquire OCT images (~5 min) and Raman spectra (<20 min) for each sample with the current approach. These times are comparable to the fine-needle aspiration biopsy (Figure 1) routinely performed in veterinary clinics. However, our automated approach requires less qualified personnel since only the measurements are performed manually. This makes the method attractive for real-time usage directly after the sample excision. Raman spectra acquisition time ex vivo could be further reduced by decreasing the integration time and increasing the incident light intensity, as shown by Dantas et al. [14], who acquired Raman spectra of malignant canine mammary lesions with an integration time of 10 s.

Artificial intelligence and advanced imaging methods are gaining popularity in veterinary medicine [44]. Our study is one of the first to introduce advanced imaging methods to veterinary oncology since no other veterinary research has attempted the multimodal approach for differentiating benign and malignant skin tumors. Thus far, only a few studies have used artificial intelligence for image analysis or studying animal health in general [45,46].

## 4. Conclusions

In conclusion, our algorithm extracted statistically significant features from OCT images and Raman spectra. LDA supervised classification proved to be an appropriate method to discriminate between malignant and benign tumors. Combining OCT and Raman data, a multimodal approach resulted in an increased class separation: skin, lipomas, and malignant tissue were differentiated with sensitivities of 0.968, 1, and 0.939, respectively, and specificities of 0.956, 1, and 0.977, respectively. These results showed the potential of a multimodal approach to detect ex vivo cancers in companion animals. However, in vivo implementation of our approach together with veterinary clinics should be a future goal.

## Figures and Tables

**Table 1:** Significance between average Raman spectral band intensity values (***, **, *—significant, NS—nonsignificant).

Raman Shift (cm^−1^)	925–970 (p1)	1060–1160 (p2)	1180–1280 (p3)	1310–1380 (p4)	1451–1509 (p5)	1845–1880 (p6)	2050–2080 (p7)	Total Average (p8)
MCT vs. STS	NS	NS	NS	NS	NS	NS	NS	***
MCT vs. lipoma	***	**	***	***	***	***	***	***
MCT vs. skin	***	NS	NS	NS	***	*	NS	***
STS vs. lipoma	***	**	***	***	***	***	***	***
STS vs. skin	***	NS	NS	NS	***	***	NS	***
lipoma vs. skin	***	**	***	**	**	***	***	***

**Table 2:** Sensitivities and specificities for skin, lipoma, MCT, STS, and malignant (MCT+STS) tissue class. Results are based on Raman, OCT, and combined datasets.

	Skin	Lipoma	MCT	STS	Malignant (MCT + STS)
Raman					
sensitivity	0.935	1	0.571	0.652	0.818
specificity	0.887	1	0.939	0.895	0.953
OCT					
sensitivity	0.839	0.75	0.5	0.739	0.879
specificity	0.857	1	0.909	0.86	0.907
Raman + OCT (combined)					
sensitivity	0.968	1	0.786	0.737	0.939
specificity	0.956	1	0.935	0.947	0.977

## Data Availability

Data that support the findings of this study are available upon request from the authors.

## Supplementary Materials

The following supporting information can be downloaded at: https://www.mdpi.com/article/10.3390/cancers14122820/s1, Table S1: Information about the tumor samples collected.

## Abbreviations

CV: coefficient of variation, FNC: fine-needle cytology, LDA: linear discriminant analysis, MCT: mast cell tumor, OCT: optical coherence tomography, STS: soft tissue sarcoma.
