# Supplementary material for: Multimodal Approach of Optical Coherence Tomography and Raman Spectroscopy Can Improve Differentiating Benign and Malignant Skin Tumors in Animal Patients
Source: Cancers (Basel). 2022 Jun 7;14(12):2820. doi: 10.3390/cancers14122820 (PMC9221378; doi:10.3390/cancers14122820)
Supplement: Supplementary file 1 [file cancers-14-02820-s001.zip › cancers-1745007-supplementary.pdf]

Table S1
Information about the tumor samples collected

#	NUMBER OF SAMPLE	SPECIES	BREED	AGE	TUMOUR	GRADE OF MALIGNANCY	LOCALIZATION
1	20B/0273	dog	chi-hua-hua	3 y/o	lipoma	-	subcutis, shoulders
2	20B/0383	cat	sphynx	7 y/o	lipoma	-	subcutis, hind leg
3	20B/0402	cat	mix	12 y/o	fibrolipoma	-	subcutis, hind leg
4	20B/0424	dog	rottweiler	5 y/o	fibrolipoma	-	subcutis, front leg
5	20B/0509	dog	Labrador	n/a	lipoma	-	subcutis, base of tail
6	20B/0285	dog	American Staffordshire terrier	12 y/o	lipoma	-	subcutis, left axilla
7	20B0707	dog	Flandrian bouvier	11 y/o	lipoma, right flank	-	subcutis, right flank
8	20B0817	dog	rottweiler	6 y/o	lipoma	-	subcutis, mammary gland
9	20B0831	dog	American Staffordshire terrier	6 y/o	lipoma	-	subcutis, right hind leg
10	20B0845	dog	golden retriever	11 y/o	lipoma	-	subcutis
11	20B0847	dog	Labrador retriever	7 y/o	lipoma	-	subcutis, left flank
12	20B0879	dog	Jack-Russel terrier	6 y/o	lipoma	-	subcutis, left axillary region
13	20B0896	dog	long hairedcollie	7 y/o	infiltrative lipoma	-	subcutis, muscles, abdominal wall
14	20B0029	dog	French bulldog	11 y/o	MCT	high grade of malignancy	skin, preputium
15	20B0055	dog	pug	8 y/o	MCT	index of mitosis 2	subcutis, chest
16	20B0066	dog	dachshund	5 y/o	MCT	high grade of malignancy	neck
17	20B0072	dog	Jack-Russel terrier	6 y/o	MCT	low grade of malignancy	skin, scrotum
18	20B0135	cat	mix	10 y/o	MCT	n/a	skin, shoulders
19	20B0150	dog	golden retriever	3 y/o	MCT	low grade of malignancy	skin, shoulders
20	20B0270	dog	Staffordshire terrier	9 y/o	MCT	low grade of malignancy (x2);	skin, back (x2)
21	20B0310	dog	Jack-Russel terrier	11 y/o	MCT	low grade of malignancy	skin, scapular region
22	20B0318	cat	sphynx	n/a	MCT	n/a	skin, neck
23	20B0347	dog	cane corso	5 y/o	MCT	index of mitosis <1	subcutis, frontal region

24	20B0446	dog	retriever	7 y/o	MCT	low grade of malignancy; 2. grade	skin, metatarsal region
25	20B0516	dog	French bulldog	9 y/o	MCT	low grade of malignancy	skin, ear
26	20B0544	dog	American bulldog	9 y/o	MCT	low grade of malignancy	skin, hind leg
27	20B0657	cat	mix	6 y/o	MCT	low grade of malignancy	skin, mammary glands
28	20B0935	dog	American Staffordshire terrier	4 y/o	MCT	low grade of malignancy	skin, ventral part of chest
29	20B0016	cat	mix	12 y/o	STS	III	skin, base of tail
30	20B0041	cat	mix	13 y/o	anaplastic sarcoma	III	subcutis
31	20B0422	dog	cane corso	8 y/o	anaplastic sarcoma	III	skin, scapular region
32	20B0435	cat	mix	11 y/o	hemangiosarcoma	n/a	subcutis, scapular region
33	20B0489	dog	Jack-Russel terrier	10 y/o	STS	II	skin, elbow
34	20B0493	cat	Persian	11 y/o	fibrosarcoma	II	shoulder
35	20B0507	dog	mix	2 y/o	hemangiosarcoma		skin, subcutis, knee
36	20B0543	dog	mix	10 y/o	STS	II	skin, elbow
37	20B0493	cat	Persian	11 y/o	fibrosarcoma	II	shoulder
38	20B0614	dog	Labrador	3 y/o	STS	III	subcutis, elbow
39	20B0621	dog	mix	12 y/o	STS	II	hind leg
40	20B0623	cat	mix	11 y/o	STS	II	subcutis, chest
41	20B0614	dog	Labrador	3 y/o	STS	III	subcutis, elbow
42	20B0649	dog	mix	12 y/o	STS	III	subcutis, perianal region
43	20B0647	dog	mix	6 y/o	STS	II	skin, subcutis, right axillary region
44	20B0552	dog	Bernese shepherd	9 y/o	mixed liposarcoma	II	ear
45	20B0679	cat	mix	13 y/o	fibrosarcoma	III	subcutis, left hind leg
46	20B0662	dog	Russian-European laika	10 y/o	STS	II	skin, subcutis, left anconeus region
47	20B0776	cat	mix	11 y/o	fibrosarcoma	II	subcutis, between shoulders
48	20B0782	dog	mix	8 y/o	hemangiopericytoma	I	skin, subcutis, left elbow region
49	20B0835	cat	British short hair	9 y/o	fibrosarcoma	II	subcutis, between shoulders
50	20B0860	cat	mix	11 y/o	STS (peripheral nerve tumor)	II	skin, subcutis, chest region
51	20B0868	cat	mix	5 y/o	fibrosarcoma	II	subcutis, caudal part of back
